# Conjugated Polymer Films Having a Uniaxial Molecular Orientation and Network Structure Prepared by Electrochemical Polymerization in Liquid Crystals

**DOI:** 10.3390/polym13152425

**Published:** 2021-07-23

**Authors:** Jiuchao Dong, Shigeki Nimori, Hiromasa Goto

**Affiliations:** 1 Division of Materials Science, Faculty of Pure and Applied Sciences, University of Tsukuba, Tsukuba 305-8573, Japan; extra9dong@gmail.com; 2 National Institute for Materials Science, Tsukuba 305-0003, Japan; nimori.shigeki@nims.go.jp

**Keywords:** orientation, crosslink, conjugated polymers, electrochemical polymerization, liquid crystal

## Abstract

A new method for fabricating conjugated polymer films was developed using electrochemical polymerization in liquid crystals and magnetic orientation. A uniaxial main chain orientation and a crosslinked network structure were achieved with this method. By employing eight types of monomers, the influence of the crosslinking for the film was investigated. The crosslinking was found to improve the solvent resistance of the conjugated polymer films. This new method is expected to be useful in various applications, such as high-powered organic electronic devices with durability.

## 1. Introduction

Due to their unique chemical structure, conjugated polymers can demonstrate inorganic semiconductor-like properties and are thus regarded as a promising material for applications. Many researchers have contributed toward the development of organic electronic devices having conjugated polymers, such as organic field-effect transistors [1,2,3], organic photovoltaic devices [4,5], and organic light-emitting diodes [6,7,8]. Compared with inorganic semiconductor materials, conjugated polymers usually have a linear form, which provides anisotropy at the molecular level. The optical absorption, emission properties, and electrical conductivity highly depend on the molecular arrangement [9,10,11,12]. Although conjugated polymers can form a well-organized structural arrangement through self-assembly, polymer films generally form poly-domains or a random arrangement. As a result, the electronic devices based on the polymers do not exhibit anisotropic optical or electrical performances. To address this concern, many groups have developed technologies to control the arrangement of conjugated polymers [13,14,15,16]. These methods include the synthesis of liquid crystalline polymers [17,18,19,20] using mechanical force [21], and oriented substrates to fabricate conjugated polymer films with uniaxial alignment [22,23].

The durability of conjugated polymer films is important for applications in organic electronic devices [24,25]. A possible solution to this issue is to construct a network structure of conjugated polymers by crosslinking; this can help in stabilizing the morphology of the conjugated polymer film [26,27]. However, molecular orientation is somewhat difficult to achieve for polymers with a network structure. A photochemical reaction or a coordination reaction allows the polymers to have both well-organized and crosslinked forms [28,29]. However, there remains a significant technical challenge to apply this technology to other conjugated polymers.

Electrochemical polymerization is a convenient method to synthesize conjugated polymer films. After polymerization, the conjugated polymers usually separate from the electrolyte solution and are then directly deposited on the electrode substrate to form films. Many groups have investigated electrochemical polymerization in terms of its mechanism [30,31,32,33] and applications, including chemical sensors [34,35], electrochromic materials [36], electroluminescent materials [37,38], and organic photovoltaic devices [39]. Our group developed a new electrochemical polymerization in a liquid crystal (LC) electrolyte solution. In contrast to traditional isotropic solvents, when LC solvents are used as the reaction medium for electrochemical polymerization, the resultant conjugated polymers tend to align along the director of the liquid crystals (LCs). The polymer films prepared in the LC have a molecular order, which is similar to that of the LCs. When smectic A LCs were employed, polarized optical microscopy (POM) observations indicated that the resulting conjugated polymer films showed a fan-shaped birefringence texture, which is the typical optical texture of smectic A LCs [40]. When cholesteric LCs were employed for the polymerization, the resulting films had an intermolecular helical order and showed a fingerprint texture similar to that of cholesteric LCs with circular dichroism optical absorption [41,42,43,44]. The most significant aspect of this preparation method is the alignment of the conjugated polymers, which can be modified by adjusting the orientation of the host LC molecules. Conjugated polymer films having a unidirectionally oriented molecular alignment were fabricated using a magnetic field [45]. We found a new way to fabricate conjugated polymer films whose molecular orientation can be controlled with a magnetic field in the polymerization process.

In this study, using electrochemical polymerization in LCs, a new method was developed to fabricate conjugated polymer films having a uniaxial molecular orientation and a crosslinked network structure. Both the uniaxial molecular orientation and preparation of crosslinked network structures allow the conjugated polymer films to show anisotropic optical properties and durability, which may endow organic electronic devices with high performance. To investigate the influence of crosslinking, eight types of monomers were employed in the synthesis for the production of crosslinked and uncrosslinked polymers. Electrochemical polymerization was conducted in a 12 T magnetic field generated by a superconducting magnet to ensure occurrence of the polymerization in an LC reaction medium with orientation. The resulting conjugated polymer films were characterized by POM observations and linear dichroism (LD) UV–vis spectroscopy to investigate the following issues: (1) uniaxial orientation, (2) orientation degree of the polymers, and (3) optical properties of the films.

## 2. Experimental

### 2.1. Materials

**Scheme 1:**
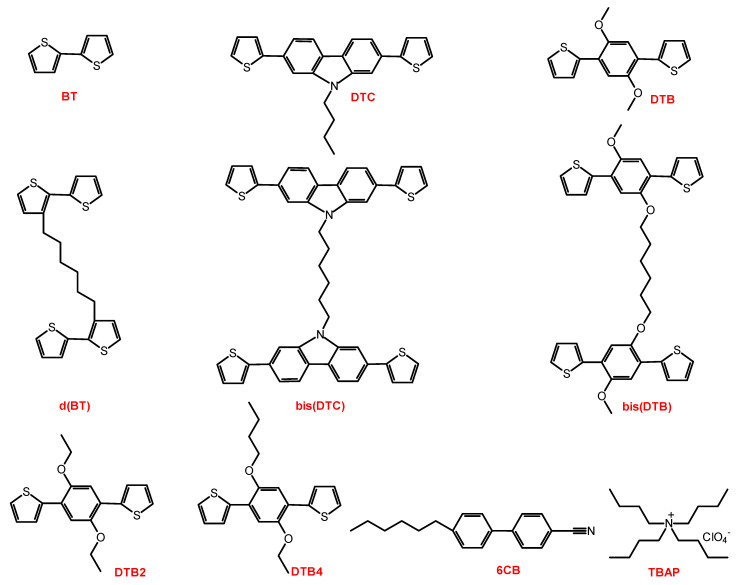
Monomers employed for the electrochemical polymerization in liquid crystals, and molecular structures of liquid crystal 4-cyano-4′-hexylbiphenyl (6CB) and the supporting salt tetrabutylammonium perchlorate (TBAP).

**Scheme 2:**
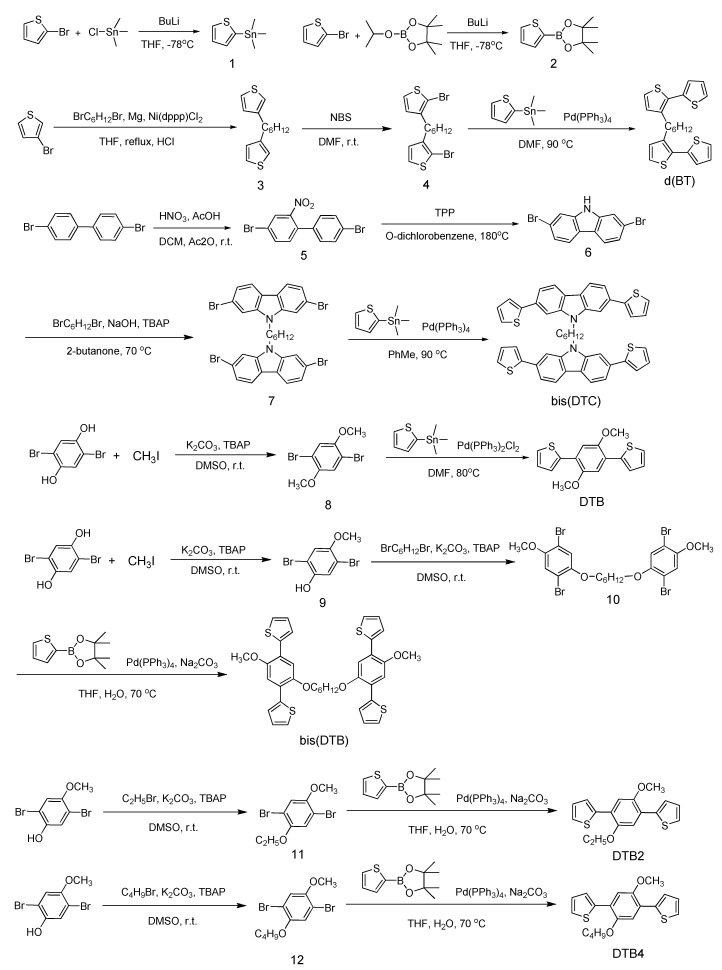
Synthesis for monomers d(BT), bis(DTC), DTB, bis(DTB), DTB2, and DTB4.

Eight types of monomer were used, including 2,2′-bithiophene (**BT**), 1,6-di(2,2′-bithiophen-3-yl)hexane (**d(BT)**), 9-butyl-2,7-di(thiophen-2-yl)-9*H*-carbazole (**DTC**), 1,6-bis(2,7-di(thiophen-2-yl)-9*H*-carbazol-9-yl)hexane (**bis(DTC))**, 1,4-dimethoxy-2,5-di(thiophen-2-yl)benzene (**DTB**), 1,6-bis(4-methoxy-2,5-di(thiophen-2-yl)phenoxy)hexane (**bis(DTB)**), 1-ethoxy-4-methoxy-2,5-di(thiophen-2-yl)benzene (**DTB2**), and 1-butoxy-4-methoxy-2,5-di(thiophen-2-yl)benzene (**DTB4**), and they are shown in Scheme 1. In addition, the matrix LC 4-cyano-4′-hexylbiphenyl (**6CB**) and the supporting salt tetrabutylammonium perchlorate (**TBAP**) are shown in Scheme 1. The syntheses of the monomers **d(BT)**, **bis(DTC)**, **DTB**, **bis(DTB)**, **DTB2**, and **DTB4** are illustrated in Scheme 2. The synthesis of **DTC** was reported in our previous work [46]. The monomer **BT** and the supporting salt **TBAP** were purchased from Tokyo Chemical Industry (TCI). The LC **6CB** was purchased from Merck. All other necessary chemicals were purchased from TCI, Wako Pure Chemical, Nacalai Tesque, and Sigma-Aldrich.

### 2.2. Synthesis

#### 2.2.1. Trimethyl(thiophen-2-yl)stannane (**1**)

First, 2-bromothiophene (1.63 g, 10 mmol) was dissolved in dry tetrahydrofuran (THF, 35 mL) and cooled to −78 °C. A solution of *n*-butyllithium in hexane (6.88 mL, 11 mmol) was added dropwise to the mixture and stirred for 1.5 h at −78 °C. Then, trimethyltinchloride (2.39 g, 12 mmol) was added to the solution at −78 °C and the mixture was allowed to slowly warm to room temperature followed by stirring for 24 h. Then, dichloromethane (DCM) was used to extract the product. The combined organic extracts were dried using Na_2_SO_4_, followed by filtration and evaporation to yield **1** as a yellow liquid (2.16 g, 8.75 mmol, 87.5%).

^1^H NMR (400 MHz; CDCl_3_; TMS) *δ* (ppm) 0.376 (s, 9H), 7.223–7.273 (m, 2H), 7.649 (d, 1H, *J* = 5.6 Hz). ^13^C NMR (100 MHz; CDCl_3;_ TMS) *δ* (ppm) 127.895, 130.716, 134.948 137.064.

#### 2.2.2. 4,4,5,5-Tetramethyl-2-(thiophen-2-yl)-1,3,2-dioxaborolane (**2**)

2-Bromothiophene (1.63 g, 10 mmol) was dissolved in dry THF (35 mL) and cooled down to −78 °C. A solution of *n*-butyllithium in hexanes (6.56 mL, 10.5 mmol) was added dropwise, and the mixture was stirred for 2 h at −78 °C. 2-Isopropoxy-4,4,5,5-tetramethyl-1,3,2-dioxaborolane (2.05 g, 11 mmol) was subsequently added at −78 °C, and the mixture was allowed to slowly warm up to room temperature and stirred for 22 h. After the reaction, the mixture was extracted with DCM. The combined organic extracts were dried by MgSO_4_, followed by filtration, and was purified by silica gel column chromatography (eluent: hexane/ethyl acetate = 9/1) to yield a yellow liquid (1.04 g, 4.96 mmol, Y = 49.6%).

^1^H NMR (400 MHz; CDCl_3_; TMS) *δ* (ppm) 1.278 (s, 12H), 7.108–7.128 (dd, 1H, *J* = 3.2, 4.4 Hz), 7.552–7.576 (dd, 1H, *J* = 1.2, 8.8 Hz), 7.566–7.581 (d, 1H, *J* = 6.0 Hz). ^13^C NMR (100 MHz; CDCl_3;_ TMS) *δ* (ppm) 24.828, 84.081, 115.905, 128.210, 132.356, 137.140.

#### 2.2.3. 1,6-Di(thiophen-3-yl)hexane (**3**)

1,6-Bromohexane (2.44 g, 10 mmol) was slowly added to a suspension of magnesium turnings (0.583 g, 24 mmol) in 25 mL of anhydrous THF and a spot of iodine followed by reflux for 3 h. 3-Bromothiophene (3.42 g, 21 mmol) and dichloro[1,3-bis(diphenylphosphino)propane]nickel (0.054 g, 0.1 mmol) was dissolved in 20 mL of THF. Then the Grignard solution was transferred via injection syringe and added dropwise to this solution at 0 °C. The resulting mixture was stirred at room temperature for 15 h, hydrolyzed with 1 M of HCl (14 mL) at 0 °C, followed by extraction with DCM. The combined organic extracts were dried over MgSO_4_ and purified by silica gel column chromatography (eluent: hexane), followed by drying, to give a colorless liquid (2.5 g, 10 mmol, Y = 100%).

^1^H NMR (400 MHz; CDCl_3_; TMS) *δ* (ppm) 1.342 (quint, 4H, *J* = 3.8 Hz), 1.583 (quint, 4H, *J* = 7.5 Hz), 2.595 (t, 4H, *J* = 7.6), 6.927–6.896 (m, 4H), 7.215–7.236 (dd, 2H, *J* = 3.2, 5.2 Hz). ^13^C NMR (100 MHz; CDCl_3_; TMS) *δ* (ppm) 29.069, 30.241, 30.479, 119.784, 125.017, 128.229, 143.088.

#### 2.2.4. 1,6-Bis(2-bromothiophen-3-yl)hexane (**4**)

To a solution of 1,6-di(thiophen-3-yl)hexane (2.50 g, 10 mmol) in dimethylformamide (DMF) (20 mL), *N*-bromosuccinimide (4.9 g, 23 mmol) was slowly added at room temperature. The mixture was stirred at room temperature for 2 days. After the reaction, the mixture was extracted with ether and dried over MgSO_4_, followed by purification by silica gel column chromatography (eluent: hexane) to yield **4** as a yellow oil (1.274 g, 3.12 mmol, Y = 31.2%).

^1^H NMR (400 MHz; CDCl_3_; TMS) *δ* (ppm) 1.341 (quint, 4H, *J* = 3.7 Hz), 1.538 (quint, 4H, *J* = 7.3 Hz), 2.536 (t, 4H, *J* = 7.6 Hz), 6.768 (d, 2H, *J* = 5.2 Hz), 7.166 (d, 2H, *J* = 6.0 Hz). ^13^C NMR (100 MHz; CDCl_3_; TMS) *δ* (ppm) 28.907, 29.345, 29.641, 108.862, 125.179, 128.191, 141.782.

#### 2.2.5. 1,6-Di(2,2′-bithiophen-3-yl)hexane (**d(BT)**)

Under an argon atmosphere, 1,6-bis(2-bromothiphen-3-yl)hexane (0.163 g, 0.4 mmol) and trimethyl(thiophen-2-yl)stannane (0.247, 1 mmol) were weighed into a two-neck flask and dissolved in 2 mL of DMF, followed by degassing for 10 min. Then, tetrakis(triphenylphosphine)palladium(0) (0.034 g, 0.03 mmol) was added. After stirring at 90 °C for 22 h, followed by cooling down, the solution was extracted with chloroform and dried over MgSO_4_. The product was purified by silica gel column chromatography (eluent: hexane/ethyl acetate = 47/3) to yield **d(BT)** as a green oil (0.122 g, 0.29 mmol, Y = 73%).

^1^H NMR (400Hz; DMSO; TMS) *δ* (ppm) 1.345 (quint, 4H, *J* = 3.5 Hz), 1.576 (quint, 4H, *J* = 7.3 Hz), 2.701 (t, 4H, *J* = 8.0 Hz), 6.897 (d, 2H, *J* = 5.6 Hz), 7.030 (m, 2H), 7.080 (d, 2H, *J* = 3.6 Hz), 7.149 (d, 2H, *J* = 5.6 Hz), 7.272 (d, 2H, *J* = 5.2 Hz). ^13^C NMR (100Hz; DMSO; TMS) *δ* (ppm) 29.021, 30.327, 34.235, 123.730, 125.274, 125.493, 125.999, 127.285, 129.840, 136.140, 139.504.

#### 2.2.6. 4,4′-Dibromo-2-nitro-biphenyl (**5**)

Under an argon atmosphere, 4,4′-dibromobiphenyl (3.12 g, 10 mmol) was weighed into a two-neck flask and dissolved in 10 mL of DCM and 30 mL of acetic anhydride. Then, 5 mL of acetic acid and 9.3 mL of nitric acid were added dropwise with an ice bath. After addition, the mixture was allowed to warm up to room temperature. After stirring for 3 h, the solution was extracted with DCM and dried over MgSO_4_. The solvent was removed on a rotary evaporator, and the residue was purified by silica gel column chromatography, (eluent: hexane/ethyl acetate = 4/1), followed by evaporation, to yield **5** as a yellow solid (1.84 g, 5.15 mmol, Y = 51%).

^1^H NMR (400Hz; CDCl_3_; TMS) *δ* (ppm) 7.142–7.160 (d, 2H, *J* = 7.2 Hz), 7.272–7.293 (d, 1H, *J* = 8.4 Hz), 7.547–7.568 (d, 2H, *J* = 8.4 Hz), 7.736–7.763 (dd, 1H, *J* = 8.0, 2.8 Hz), 8.022–8.027 (d, 1H, *J* = 2.0 Hz). ^13^C NMR (100Hz; CDCl_3_; TMS) *δ* (ppm) 100.179, 121.862, 123.006, 127.323, 129.439, 131.994, 132.966, 134.205, 135.434, 163.817,172,624.

#### 2.2.7. 2,7-Dibromo-9H-carbazole (**6**)

Under an argon atmosphere, 4,4′-dibromo-2-nitro-biphenyl (1.84 g, 5.15 mmol) and triphenylphosphine (4.05 g, 15.5 mmol) were weighed into a two-neck flask and dissolved with 15 mL of *o*-dichlorobenzene. The mixture was heated to 180 °C. After stirring for 19 h, the solvent was removed with a rotary evaporator, and the residue was purified by silica gel (eluent: hexane/chloroform = 3/1) followed by evaporation to yield **6** as a brown solid (1.29 g, 3,97 mmol, Y = 77%).

^1^H NMR (400Hz; CDCl_3_; TMS) *δ* (ppm) 7.336–7.360 (dd, 2H, *J* = 8.0, 1.2 Hz), 7.563–7.568 (d, 2H, *J* = 2.0 Hz), 7.856–7.877 (d, 2H, *J* = 8.4 Hz), 8.056 (s, 1H). ^13^C NMR (100Hz; CDCl_3_; TMS) *δ* (ppm) 113.789, 119.708, 121.443, 121.738, 123.263, 140.228.

#### 2.2.8. 1,6-Bis(2,7-dibromo-9H-carbazol-9yl)hexane (**7**)

Under an argon atmosphere, 2,7-dibromo-9*H*-carbazole (0.325 g, 1 mmol), 1,6-dibromohexane (0.122 g, 0.5 mmol), sodium hydroxide (0.08 g, 2 mmol), and tetrabutylammonium perchlorate (0.014 g, 0.04 mmol) were weighed into a two-neck flask and dissolved in 3 mL of 2-butanone. The mixture was heated to 70 °C. After stirring for 39 h, the organic layer was extracted with chloroform, and then dried by MgSO_4_ followed by filtration. After evaporation, the residue was collected by recrystallization in chloroform to yield **7** as a brown solid (0.292 g, 0.4 mmol, Y = 80%).

^1^H NMR (400Hz; CDCl_3_; TMS) *δ* (ppm) 1.429 (m, 4H), 1.825 (m, 4H), 4.144–4.179 (t, 4H, *J* = 7.0 Hz), 7.304–7.325 (d, 4H, *J* = 8.4 Hz), 7.468 (s, 4H), 7.841–7.863 (d, 4H, *J* = 8.8 Hz).

#### 2.2.9. 1,6-Bis(2,7-di(thiophen-2-yl)-9H-carbazol-9-yl)hexane (**bis(DTC)**)

Under an argon atmosphere, 1,6-bis(2,7-dibromo-9*H*-carbazol-9-yl)hexane (0.146 g, 0.2 mmol) and trimethyl(thiophen-2-yl)stannane (0.247, 1 mmol) were weighed into a two-neck flask and dissolved in 2 mL of toluene and 2 mL of THF, followed by degassing for 10 min. Then, tetrakis(triphenylphosphine)palladium(0) (0.034 g, 0.03 mmol) was added. After stirring at 90 °C for 4 days, followed by cooling down, the solution was extracted with chloroform and dried over MgSO_4_. The product was purified by recrystallization in chloroform/methanol, and column chromatography (hexane/chloroform = 2/1) was conducted to yield **bis(DTC)** as a green solid (0.032 g, 0.045 mmol, Y = 22.5%).

^1^H NMR (400Hz; DMSO; TMS) *δ* (ppm) 1.514 (m, 4H), 1.904 (m, 4H), 4.367–4.401 (t, 4H, *J* = 6.8 Hz), 7.078–7.098 (m, 4H), 7.306–7.319 (d, 4H, *J* = 5.2 Hz), 7.420–7.458 (m, 8H), 7.607 (s, 4H), 8.001–8.022 (d, 4H, *J* = 8.4 Hz).

#### 2.2.10. 1,4-Dibromo-2,5-bis-methoxybenzene (**8**)

2,5-Dibromohydroquinone (2.679 g, 10 mmol), tetrabutylammonium perchlorate (0.171 g, 0.5 mmol), and potassium carbonate (4.15 g, 30 mmol) were mixed in acetone (30 mL). Iodomethane (4.26 g, 30 mmol) was added to the solution. Then, the mixture was stirred at room temperature for 24 h. The mixture was extracted with DCM twice, and the organic layer was dried over MgSO_4_. Purification with silica gel column chromatography (eluent: chloroform) gave **8** as a white solid (0.949 g, 34 mmol, Y = 34%).

^1^H NMR (600 MHz; CDCl_3_; TMS) *δ* (ppm) 3.851 (s, 6H), 7.105 (s, 2H).

#### 2.2.11. 1,4-Dimethoxy-2,5-di(thiophen-2-yl)benzene (DTB)

Under an argon atmosphere, 1,4-dibromo-2,5-dimethoxybenzene (0.296 g, 1 mmol) and trimethyl(thiophen-2-yl)stannane (0.519, 2 mmol) were weighed into a two-neck flask and dissolved in 5 mL of DMF, followed by degassing for 10 min. Then, bis(triphenylphosphine)palladium(II)dichloride (0.048 g, 0.12 mmol) was added. After stirring at 80 °C for 24 h, followed by cooling down, the solution was extracted with DCM and dried over MgSO_4_. The solvent was removed with a rotary evaporator and the residue was purified by silica gel column chromatography (eluent: hexane/chloroform = 2.5/1), followed by evaporation, to yield **DTB** as a green solid (0.121 g, 0.4 mmol, Y = 40%).

^1^H NMR (400Hz; CDCl_3_; TMS) *δ* (ppm) 3.941 (s, 6H), 7.089–7.111 (dd, 2H, *J* = 5.2, 3.2 Hz), 7.247 (s, 2H), 7.333–7.348 (dd, 2H, *J* = 5.2, 1.6 Hz), 7.520–7.532 (dd, 2H, *J* = 3.6, 2.4 Hz). ^13^C NMR (100Hz; CDCl_3_; TMS) *δ* (ppm) 56.442, 112.331, 122.987, 125.474, 125.694, 126.904, 139.037, 149.831.

#### 2.2.12. 2,5-Dibromo-4-methoxyphenol (**9**)

2,5-Dibromohydroquinone (2.14 g, 8 mmol), tetrabutylammonium perchlorate (0.034 g, 0.1 mmol), and potassium carbonate (1.38 g, 10 mmol) were mixed in dimethyl sulfoxide (DMSO, 15 mL). Iodomethane (1.135 g, 8 mmol) was added to the solution dropwise. Then, the mixture was stirred at room temperature for 16 h. The reaction mixture was extracted with DCM and dried over magnesium sulfate. Purification by silica gel column chromatography (eluent: hexane/ethyl acetate = 4/1) gave **9** as a white solid (0.784 g, 2.78 mmol, Y = 35%).

^1^H NMR (400 MHz; CDCl_3_; TMS) *δ* (ppm) 3.836 (s, 3H), 5.146 (s, 1H), 6.979 (s, 1H), 7.247 (s, 1H). ^13^C NMR (100 MHz; CDCl_3_; TMS) *δ* (ppm) 57.128, 108.376, 111.817, 115.181, 120.413, 146.824, 150.341.

#### 2.2.13. 1,6-Bis(2,5-dibromo-4-methoxyphenoxy)hexane (**10**)

2,5-Dibromo-4-methoxyphenol (0.564 g, 2 mmol), tetrabutylammonium perchlorate (0.0085 g, 0.025 mmol), and potassium carbonate (0.345 g, 2.5 mmol) were mixed in DMSO (8 mL). 1,6-Dibromohexane (0.232 g, 0.95 mmol) was added to the solution dropwise. Then, the mixture was stirred at room temperature for 3 days. The mixture was extracted with DCM and dried over magnesium sulfate. Purification by silica gel column chromatography (eluent: hexane/ethyl acetate = 9/1) gave **10** as a pink solid (0.414 g, 0.637 mmol, Y = 64%).

^1^H NMR (400 MHz; CDCl_3_; TMS) *δ* (ppm) 1.570 (quint, 4H, *J* = 3.6 Hz), 1.817 (quint, 4H, *J* = 6.5 Hz), 3.840 (s, 6H), 3.956 (t, 4H, *J* = 6.2 Hz), 7.076 (s, 2H), 7.088 (s, 2H). ^13^C NMR (100 MHz; CDCl_3_; TMS) *δ* (ppm) 25.475, 28.964, 57.023, 69.976, 110.349, 111.187, 116.925, 118.517, 150.017, 150.426.

#### 2.2.14. 1,6-Bis(4-methoxy-2,5-di(thiophen-2-yl)phenoxy)hexane (**bis(DTB)**)

In an argon-flushed two-neck round-bottom flask, 4,4,5,5-tetramethyl-2-(thiophen-2-yl)1,3,2-dioxaborolane (0.21 g, 1 mmol) and 1,6-bis(2,5-dibromo-4-methoxyphenoxy)hexane (0.155 g, 0.24 mmol) were dissolved in THF (3 mL) and degassed for 10 min. Then, tetrakis(triphenylphosphine)palladium (0.069 g, 0.06 mmol) and 2 mL of the degassed Na_2_CO_3_ (0.318 g, 3 mmol)/water solution were added. The mixture was heated at 70 °C and refluxed for 24 h. After cooling down, the organic layer was extracted with DCM and dried over MgSO_4_. The solvent was removed with a rotary evaporator, and the residue was purified by column chromatography (eluent: hexane/ethyl acetate/chloroform = 4/1/1.5) to yield **bis(DTB)** as a yellow solid (0.111 g, 0.169 mmol, Y = 70%).

^1^H NMR (400Hz; CDCl_3_; TMS) *δ* (ppm) 1.624 (quint, 4H, *J* = 3.8 Hz), 1.916 (quint, 4H, *J* = 6.6 Hz), 3.935 (s, 6H), 4.081 (t, 4H, *J* = 6.4 Hz), 7.054 (t, 2H, *J* = 7.0 Hz), 7.063 (dd, 2H, *J* = 1.2, 8.8 Hz), 7.222 (s, 2H), 7.248 (s, 2H), 7.286–7.335 (m, 4H), 7.498–7.528 (m, 4H). ^13^C NMR (100Hz; CDCl_3_ TMS) *δ* (ppm) 25.990, 29.288, 56.423, 69.442, 112.064, 112.941, 122.901, 122.987, 125.150, 125.417, 125.636, 125.770, 126.685, 126.856, 139.085, 149.283, 149.788.

#### 2.2.15. 1,4-Dibromo-2-ethoxy-5-methoxybenzene (**11**)

2,5-Dibromo-4-methoxyphenol (0.226 g, 0.8 mmol), tetrabutylammonium perchlorate (0.01 g, 0.03 mmol), and potassium carbonate (0.138 g, 1 mmol) were mixed in DMSO (3 mL). Bromoethane (0.075 mL, 1 mmol) was added dropwise. Then, the mixture was stirred at room temperature for 24 h. The mixture was extracted with DCM and dried over MgSO_4_. Purification by silica gel column chromatography (eluent: hexane/ethyl acetate = 4/1) gave **11** as a white solid (0.25 g, 0.8 mmol, Y = 100%).

^1^H NMR (400 MHz; CDCl_3_; TMS) *δ* (ppm) 1.425–1.460 (t, 3H, *J* = 8.2 Hz), 3.844 (s, 3H), 4.010–4.062 (q, 2H, *J* = 6.9 Hz), 7.092 (s, 1H), 7.102 (s, 1H). ^13^C NMR (100 MHz; CDCl_3_; TMS) *δ* (ppm) 14.763, 57.004, 66.011, 110.387, 111.321, 116.982, 118.812, 149.950, 150.541.

#### 2.2.16. 2,2′-(2-Ethoxy-5-methoxy-1,4-phenylene)dithiophene (**DTB2**)

In an argon-flushed two-neck round-bottom flask, 4,4,5,5-tetramethyl-2-(thiophen-2-yl)1,3,2-dioxaborolane (0.146 g, 0.7 mmol) and 1,4-dibromo-2-ethoxy-5-methoxybenzene (0.093 g, 0.3 mmol) were dissolved in THF (3 mL) and degassed for 10 min. Then tetrakis(triphenylphosphine)palladium (0.048 g, 0.042 mmol) and 2 mL of the degassed solution of Na_2_CO_3_ (0.223 g, 2.1 mmol) in water were added. The mixture was heated at 70 °C and refluxed for 24 h. After cooling down, the organic layer was extracted with DCM and dried over MgSO_4_. The solvent was removed with a rotary evaporator, and the residue was purified by column chromatography (eluent: hexane/ethyl acetate = 4/1) to yield **DTB2** as a yellow solid (0.082 g, 0.26 mmol, Y = 88%).

^1^H NMR (400Hz; CDCl_3_; TMS) *δ* (ppm) 1.504–1.539 (t, 3H, *J* = 7.0 Hz), 3.939 (s, 3H), 4.133–4.185 (q, 2H, *J* = 6.9 Hz), 7.085–7.106 (m, 2H), 7.241–7.261 (d, 2H, *J* = 8.0 Hz), 7.326–7.340 (dd, 2H, *J* = 4.0, 5.6 Hz), 7.504–7.515 (dd, 1H, *J* = 3.6, 4.4 Hz), 7.549–7.558 (d, 1H, *J* = 3.6 Hz). ^13^C NMR (100Hz; CDCl_3_; TMS) *δ* (ppm) 15.020, 56.413, 65.420, 86.903, 112.017, 113.427, 122.920, 123.187, 125.198, 125.408, 125.627, 125.722, 126.742, 126.875, 139.066, 139.199, 149.178, 149.921.

#### 2.2.17. 1,4-Dibromo-2-butoxy-5-methoxybenzene (**12**)

2,5-Dibromo-4-methoxyphenol (0.226 g, 0.8 mmol), tetrabutylammonium perchlorate (0.01 g, 0.03 mmol), and potassium carbonate (0.138 g, 1 mmol) were mixed in DMSO (3 mL). Bromobutane (0.108 mL, 1 mmol) was added dropwise. Then, the mixture was stirred at room temperature for 24 h. The mixture was extracted with DCM and dried over magnesium sulfate. Purification by silica gel column chromatography (eluent: hexane/ethyl acetate = 4/1) gave **12** as a yellow oil (0.27 g, 0.8 mmol, Y = 100%).

^1^H NMR (400 MHz; CDCl_3_; TMS) *δ* (ppm) 0.964–1.001 (t, 3H, *J* = 7.4 Hz), 1.474–1.568 (sext, 2H, *J* = 7.5 Hz), 1.755–1.826 (quint, 2H, *J* = 7.1 Hz), 3.840 (s, 3H), 3.942–3.974 (t, 2H, *J* = 6.4 Hz), 7.087 (s, 1H), 7.094 (s, 1H). ^13^C NMR (100 MHz; CDCl_3_; TMS) *δ* (ppm) 13.829, 19.233, 31.204, 57.023, 70.023, 110.368, 11.235, 118.603, 150.131, 150.436.

#### 2.2.18. 1-Butoxy-4-methoxy-2,5-di(thiophen-2-yl)benzene (**DTB4**)

In an argon-flushed two-neck round-bottom flask, 4,4,5,5-tetramethyl-2-(thiophen-2-yl)1,3,2-dioxaborolane (0.210 g, 1 mmol) and 1,4-dibromo-2-butoxy-5-methoxybenzene (0.169 g, 0.5 mmol) were dissolved in THF (3 mL) and degassed for 10 min. Then, tetrakis(triphenylphosphine)palladium (0.069 g, 0.06 mmol) and 2 mL of the degassed solution of Na_2_CO_3_ (0.318 g, 3 mmol) in water solution were added. The mixture was heated at 70 °C and refluxed for 24 h. After cooling down, the organic layer was extracted with DCM and dried over MgSO_4_. The solvent was removed on a rotary evaporator, and the residue was purified by column chromatography (eluent: hexane/ethyl acetate = 4/1) to yield **12** as a yellow solid (0.129 g, 0.37 mmol, Y = 75%).

^1^H NMR (400Hz; CDCl_3_; TMS) *δ* (ppm) 0.981–1.018 (t, 3H, *J* = 7.4 Hz), 1.519–1.612 (sext, 2H, *J* = 7.4 Hz), 1.855–1.926 (quint, 2H, *J* = 7.1 Hz), 3.935 (s, 3H), 4.073–4.106 (t, 2H, *J* = 6.6 Hz), 7.081–7.103 (dd, 2H, *J* = 3.6, 5.2 Hz), 7.232–7.263 (d, 2H, *J* = 12.4 Hz), 7.328–7.341 (m, 2H), 7.506–7.517 (dd, 1H, *J* = 3.6, 4.4 Hz), 7.532–7.545 (d, 1H, *J* = 1.6, 3.6 Hz). ^13^C NMR (100Hz; CDCl_3_; TMS) *δ* (ppm) 13.943, 19.500, 31.480, 56.480, 69.385, 112.112, 112.989, 122.920, 123.006, 125.160, 125.389, 125.617, 125.722, 126.704, 126.866, 139.123, 139.189, 149.340, 149.778.

### 2.3. Electrochemical Polymerization in LCs

The monomers and the supporting salt (**TBAP**) were added to a nematic LC medium and mixed thoroughly to obtain an LC electrolyte solution. An indium tin oxide (ITO)-coated glass electrode was used as a substrate. For the electrochemical polymerization, the reaction cells were constructed using two ITO glass electrodes and a polytetrafluoroethylene spacer (0.20 mm thickness) to construct a sandwich structure. The LC electrolyte solution was injected into the cell, heated to the isotropic phase, and gradually cooled to room temperature. The reaction cells were placed in a magnetic field (12 T, superconducting magnet) for 20 min in order to obtain the uniaxially oriented LC reaction medium. Then, 4.0 V of direct current was applied across the cell to initiate the polymerization at room temperature. After 15 min, the power source was disconnected and the reaction cell was removed from the magnetic field. The resulting polymer film was washed with organic solvents to remove residual LC, supporting salt, and monomers. All the primary polymer films were reduced from an oxidized state to a neutral state with the treatment of hydrazine. The concentration of each component and the electrochemical polymerization conditions are summarized in Table 1.

### 2.4. Characterization Methods

^1^H NMR and ^13^C NMR spectra were recorded using a JNM-ECS spectrometer (JEOL, 400 MHz), the chemical shifts were recorded in parts per million, and the coupling constants (*J*) were recorded in Hertz. POM observations were performed using a Nikon Eclipse LV100 POM with the crossed Nicol condition. LD UV–vis absorption spectroscopy was performed using a JASCO V-630 UV–vis spectrophotometer equipped with a polarizer.

## 3. Results

### 3.1. Polymerization

**Figure 1:**
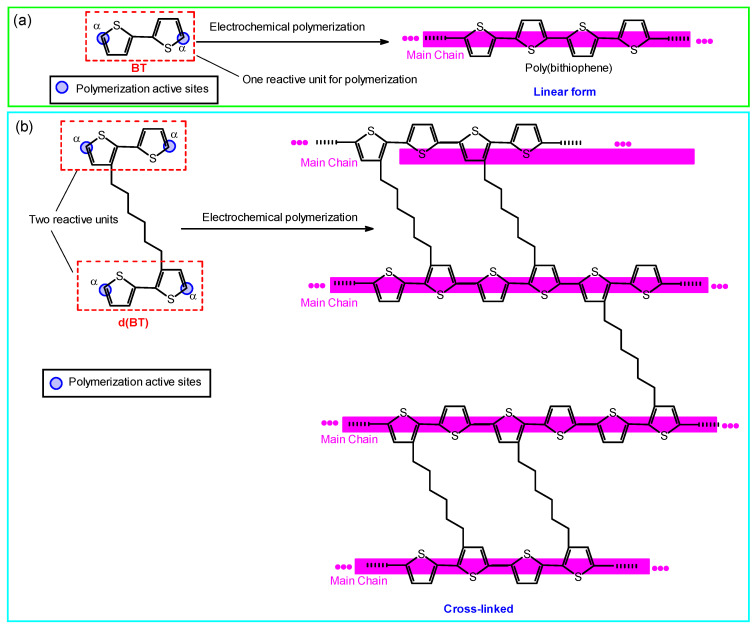
Plausible forms of linearly propagated **BT** ((**a**), uncrosslinkable) and **d(BT)** ((**b**), crosslinkable).

**BT, d(BT), DTC, bis(DTC), DTB, bis(DTB), DTB2**, and **DTB4** as monomers were used in this study. **BT, DTC**, and **DTB** have one reactive unit (conjugated backbone) with two reactive sites (α-position of the thiophene unit) in each molecule, while **d(BT), bis(DTC)**, and **bis(DTB)** have two independent reactive units with four reactive sites (α-position of the thiophene units) in each molecule. These characteristics allow **d(BT), bis(DTC)**, and **bis(DTB)** to form a crosslinked polymer structure, and copolymerization with **BT, DTC**, and **DTB** can tune the degree of crosslinking. Compared with **DTB, DTB2** and **DTB4** have the same conjugated backbone but different alkyl side chain lengths. They were used for further investigation in the examination of molecular form influence. Plausible uncrosslinked and crosslinked forms are described in the polymerization of BT and d(BT), as an example (Figure 1).

**Scheme 3:**
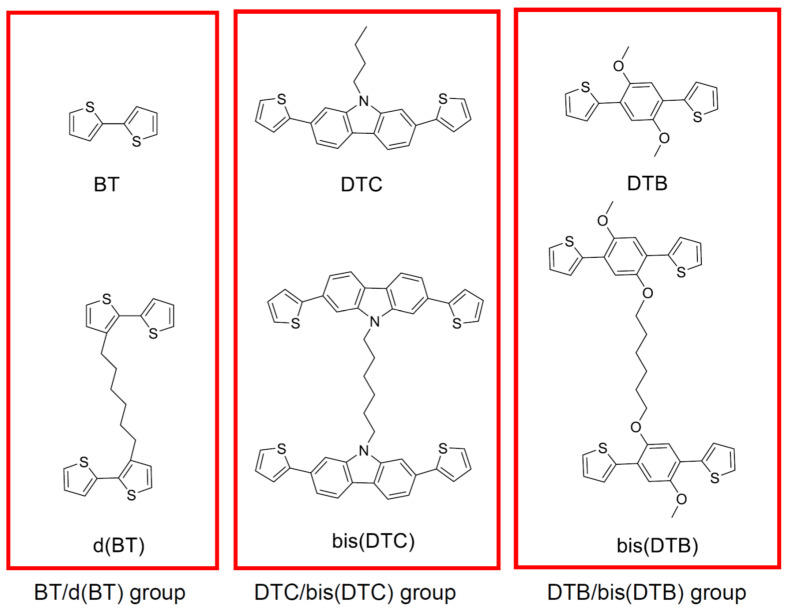
Six monomers are distributed between three groups in the experiments. The reactive unit concentration is used rather than the monomer concentration. In each electrolyte solution sample, the proportion of uncrosslinked units and crosslinked units is changed according to 10:0, 7:3, 5:5, 3:7, and 0:10 (in molar).

BT is copolymerized with d(BT), DTC is copolymerized with dis(DTC), and DTB is copolymerized with bis(DTB). Hence, we distributed these six monomers into three copolymerization groups: the **BT/d(BT)** group, the **DTC/bis(DTC)** group, and the **DTB/bis(DTB)** group (Scheme 3). Given that the uncrosslinked monomer (uncrosslinkable) (**BT**, **DTC**, and **DTB**) has one reactive unit, while the crosslink monomer (crosslinkable) (**d(BT)**, **bis(DTC)**, and **bis(DTB)**) has two reactive units, the reactive unit concentration was used rather than monomer concentration for calculation in this work. For each polymerization, the total reactive unit concentration was kept at 0.5% molar. The ratio of crosslinked units (RCU) was adjusted as 0%, 30%, 50%, 70%, and 100%, in order to examine different degrees of crosslink. Herein, RCU is employed to briefly describe the proportion of added uncrosslinked units and crosslinked units, but it should be noted that it indicates no actual degree of crosslinking in each sample.

### 3.2. POM Observation

Confirmation of birefringence is a convenient method to examine anisotropic materials. An anisotropic material possesses an optical axis. When linearly polarized light is applied to an anisotropic material, the electric vector of the light can be resolved into two vector components: one is parallel to the optical axis and the other is perpendicular to the optical axis. These two vector components of the light indicate the different propagation velocities in the material. Therefore, when these two vector components exit from the material, a phase difference between the optical waves is generated. Consequently, the electric vector of the new resulting polarized light can be differentiated from the original polarized light. This phenomenon can be described using the following equation:

Δφ = 2π|*ne* − *no*|/λ (1)

where ∆φ is the phase difference, *d* is the thickness of the sample, *λ* is the wavelength of the incident light, *ne* is the refractive index of the polarized light, where the electric vector is parallel to the optical axis, and *no* is the refractive index of the polarized light, where the electric vector is perpendicular to the optical axis. When ∆φ = (2n + 1)π, the direction of the electric vector of the emergent light is rotated 90° from the incident light; when ∆φ = 2nπ, the direction of the electric vector does not change; when 2nπ < ∆φ < (2n + 1)π, the emergent light becomes circularly polarized light or elliptically polarized light.

**Figure 2:**
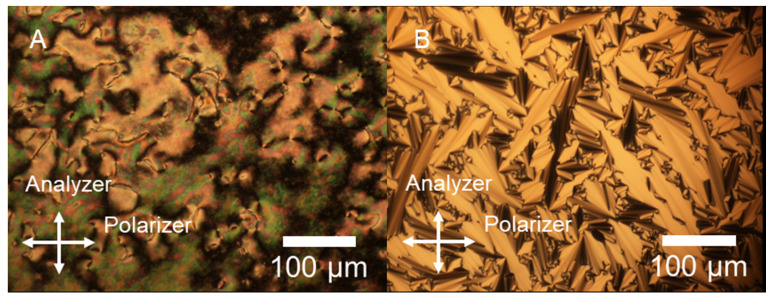
Polarized optical microscopy (POM) images of Schlieren texture of nematic liquid crystal LC (**A**) and fan-shaped texture of smectic LC (**B**).

LC materials with no orientation usually show a characteristic optical texture, such as the Schlieren texture of nematic LCs (Figure 2A) and fan-shaped texture of smectic LCs (Figure 2B). These photographs show LC textures as examples.

**Figure 3:**
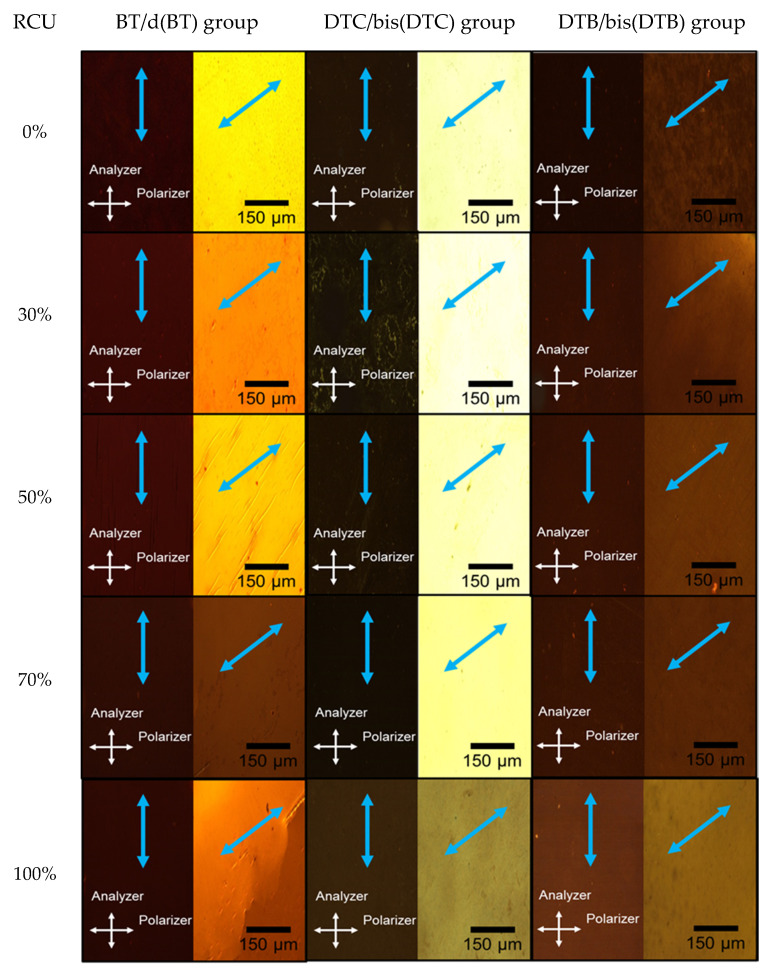
POM images of polymer films obtained from the BT/d(BT) group, DTC/bis(DTC) group, and DTB/bis(DTB) group with different RCUs. For comparison, a photograph was first taken of a sample (**left side**), and then the sample was rotated 45° and a second photograph was taken (**right side**). Blue arrows indicate the orientation direction. RCU: Ratio of crosslinked units.

Figure 3 shows the POM images of the polymer films obtained from the **BT/d(BT)** group, **DTC/bis(DTC)** group, and **DTB/bis(DTB)** group with varying RCU. The polymers were prepared in LC solution with electrochemical polymerization under the magnetic field. All samples displayed different colors and brightness levels between the left side and the right side of their pictures. When the LC orientation was parallel to the analyzer, dark pictures were observed, but when the LC orientation was not parallel to the analyzer and polarizer, significantly brighter pictures were observed. This difference was easily observed even at 100% RCU. Overall, this phenomenon indicates that all the samples were optically anisotropic, and their optical axes were parallel to the polarizer or analyzer. The conjugated polymers were uniaxially oriented even at 100% RCU.

According to Equation (1), a low orientation degree will result in a small absolute value of (*ne* − *no*), which then results in a small phase difference ∆φ. Therefore, it would be difficult to observe the emergent light using an analyzer, and changes in color or brightness between the left side and the right side of the POM image would also be imperceptible. In addition, the thickness of a sample *d* can also affect ∆φ. Therefore, the orientation degree of a polymer film cannot be evaluated only with the POM observation.

### 3.3. LD UV–Vis Absorption Spectra Measurement

**Figure 4:**
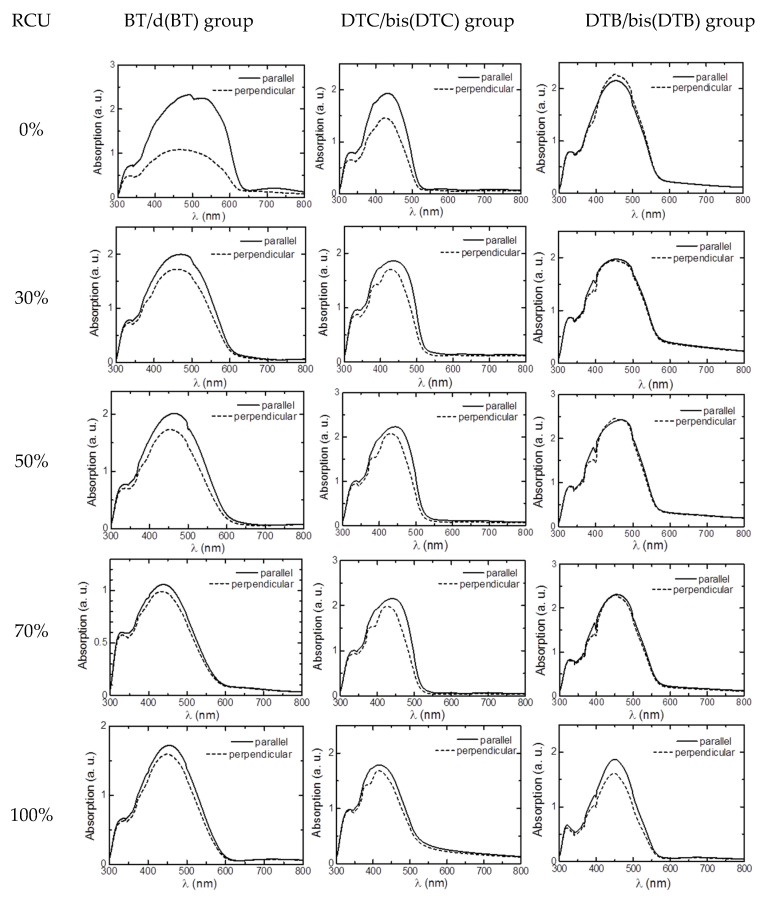
Linear dichroism (LD) UV–vis absorption spectra of polymer films obtained from the BT/d(BT) group, DTC/bis(DTC) group, and DTB/bis(DTB) group with different RCUs. In each spectrum, the solid line indicates the absorption intensity along the direction parallel to the liquid crystal orientation; the dashed line indicates the absorption intensity along the direction perpendicular to the liquid crystal orientation. LD: Linear dichroism. LDR: Linear dichroism ratio. RCU: Ratio of crosslinked units.

In linearly conjugated polymers, the transition dipole moment is along the main chain. The electronic transition probability is along the direction proportional to the inner product between the dipolar transition moment and the electric vector of the incident light. If the polarization direction of the incident light is parallel to the main chain of a conjugated polymer, the maximum absorption intensity can be observed. Therefore, the main chain orientation of conjugated polymers can be defined using linear dichroism (LD) UV–vis absorption spectroscopy. The LD measurement for every sample was carried out two times in different orientations using a polarizer. One orientation was parallel to the LC orientation and the other was perpendicular to the LC orientation. If one of the two orientations shows a higher absorption intensity than the other, it indicates the main chain orientation of the polymers. Figure 4 shows the LD UV–vis spectra of all the samples. In each spectrum, the solid line represents the absorption intensity for an orientation that is parallel to the LC orientation, and the dashed line indicates the absorption intensity for an orientation that is perpendicular to the LC orientation. In the spectra of the **BT/d(BT)** group and the **DTC/bis(DTC)** group, higher intensities were observed for the solid lines, indicating that the main chain orientation was parallel to the LC orientation. This is the parallel orientation. With an increase in the RCU, the intensity difference between the solid line and the dashed line decreased. This indicates that the increased proportion of crosslinked monomers resulted in a decrease in the orientation degree in this case.

**Figure 5:**
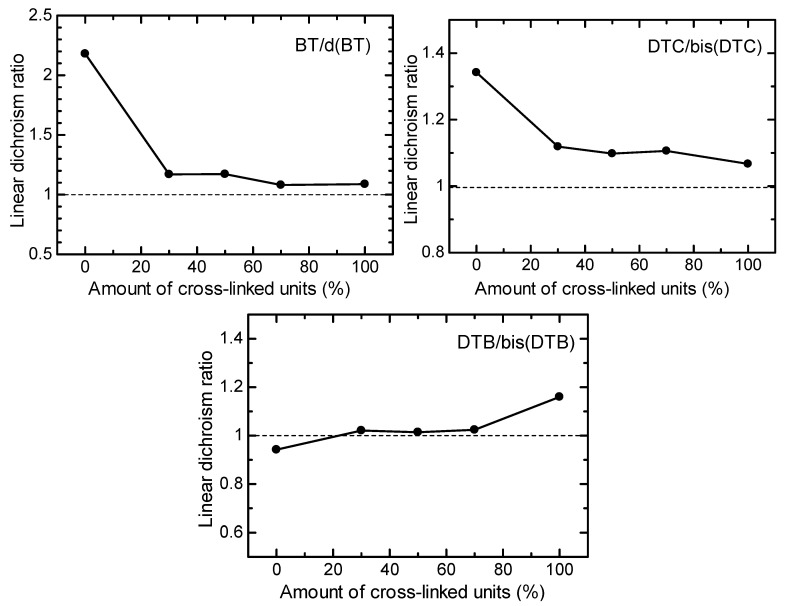
LDR of polymer films obtained from the BT/d(BT) group, DTC/bis(DTC) group, and DTB/bis(DTB) group with different RCUs. LDR: Linear dichroism ratio. RCU: Ratio of crosslinked units.

In the LD spectra of the **DTB/bis(DTB)** group, when the RCU was 0%, the dashed line showed a higher intensity, which indicates that the main chain orientation was perpendicular to the LC orientation. This is the perpendicular orientation. When the RCU was in the range of 30–70%, the solid line and the dashed line had roughly the same intensity, which suggests that the orientation degree of the polymers was low. When the RCU was 100%, the solid line gave a higher intensity, which means that the polymers were in a parallel orientation. For a better understanding of the effect of RCU on the orientation degree, a linear dichroism ratio (LDR) was summarized for all samples to evaluate the orientation degree. The LDR was calculated according to the following equation:

LDR = A_//_/A_⊥_ (2)

where A_//_ is the maximum absorption intensity of the solid line and A_⊥_ the maximum absorption intensity of the dashed line. When the LDR is greater than 1, it indicates a parallel orientation; when the LDR is smaller than 1, it indicates a perpendicular orientation; and when LDR is close to 1, it indicates that the orientation degree decreased. The results are shown in Figure 5. It is worth noting that the addition of a crosslinked monomer changed both the orientation degree and the orientation direction of the polymers in the **DTB/bis(DTB)** group. Given that 100% **DTB** resulted in a perpendicular orientation and 100% **bis(DTB)** resulted in a parallel orientation, it follows that the blending of these two monomers would result in a neutralization of the parallel orientation effect and the perpendicular orientation effect. However, it is not clear what causes the different orientation directions of **DTB** and **bis(DTB)**. It is likely that as **DTB** and **bis(DTB)** have the same conjugated backbone, the orientation difference arises due to the alkyl side chain and crosslinked structure. Therefore, the next step is to synthesize the monomers **DTB2** (with an ethyl side chain) and **DTB4** (with a butyl side chain), in order to investigate the effect of different alkyl side chains.

**Figure 6:**
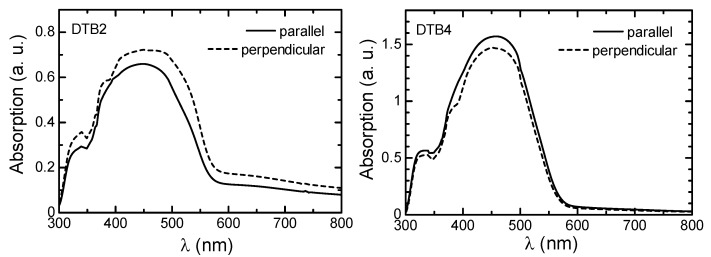
LD UV–vis absorption spectra of polymer films obtained from monomers DTB2 and DTB4. In each spectrum, the solid line indicates the absorption intensity along the direction parallel to the liquid crystal orientation; the dashed line indicates the absorption intensity along the direction perpendicular to the liquid crystal orientation.

Figure 6 shows the LD UV–vis absorption spectra of the polymer films formed using the monomers **DTB2** and **DTB4**. According to the spectra, **DTB2** was in a perpendicular orientation while **DTB4** was in a parallel orientation. This indicates that the orientation of conjugated polymers can be significantly affected with a slight change in the alkyl side chain length. It is clear that the addition of a crosslinked monomer dramatically affects the orientation degree of the polymers. In the **BT/d(BT)** and **DTC/bis(DTC)** groups, when the RCU increased to 30%, the orientation degree significantly decreased; when the RCU was increased from 30% to 100%, the orientation degree gradually decreased. In the **DTB/bis(DTB)** group, when the RCU was 0%, the polymers showed a perpendicular orientation; when the RCU was 100%, the polymers showed a parallel orientation; when the RCU was in the range of 30–70%, the orientation degree was too small to define the orientation direction.

### 3.4. Optical Properties of Polymers

**Figure 7:**
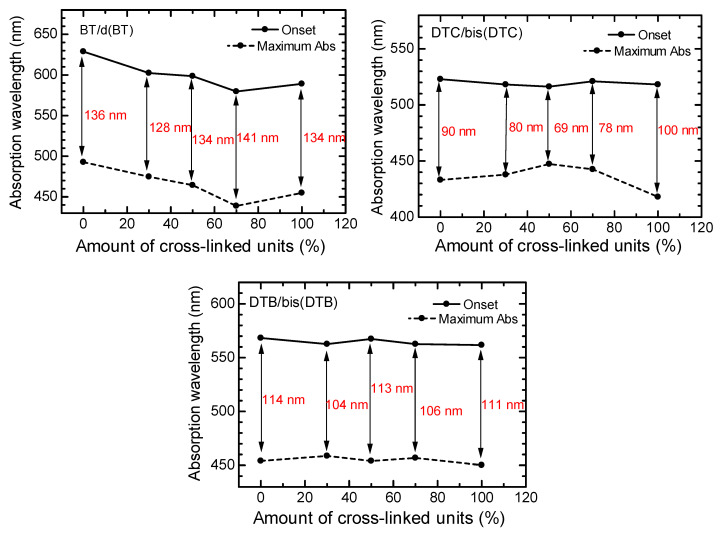
Maximum absorption wavelength and onset wavelength of polymer films synthesized from the BT/d(BT) group, DTC/bis(DTC) group, and DTB/bis(DTB) group with different RCUs. The differences between the maximum absorption wavelength and the onset wavelength are annotated in red letters.

Crosslinking affects not only the molecular orientation degree of polymers, but also their optical properties. The maximum absorption wavelength and the onset wavelength in the absorption spectra are two important parameters for evaluating the optical properties of a polymer. The maximum absorption wavelength and onset wavelength of the polymers are summarized in Figure 7. With a change in the RCU, the **BT/d(BT)**, **DTC/bis(DTC)**, and **DTB/bis(DTB)** groups showed completely different tendencies, which indicates the complexity of the crosslinking effect. For the **BT/d(BT)** group, an increased RCU resulted in a decrease in the onset and maximum absorption wavelengths; in the **DTC/bis(DTC)** group, the onset wavelength marginally changed while the maximum absorption wavelength first increased and then decreased; in the **DTB/bis(DTB)** group, the onset wavelength and maximum absorption wavelength changed in opposite directions. Given that the polymers formed within the same group have the same conjugated backbone structure, the change in the optical spectra may be due to three reasons: degree of polymerization, coplanarity of the conjugated backbone, or intermolecular interaction. When crosslinked monomers were added to the polymerization system at various concentrations, all three factors changed. It is most likely that the crosslinking effect changed the optical properties of the polymer films by affecting the molecular weight, coplanarity, and intermolecular interaction, simultaneously.

Moreover, the difference between the two parameters was calculated. In general, the absorption spectra reflect the electronic transitions between the different vibrational energy levels of the ground and excited states. The onset wavelength corresponds to the 0–0 transition and the maximum absorption wavelength corresponds to the transition with the highest transition probability. The difference between the onset wavelength and the maximum absorption wavelength can be reflected from mechanical twisting of the main chain in the excited state.

### 3.5. Solvent Resistance

**Figure 8:**
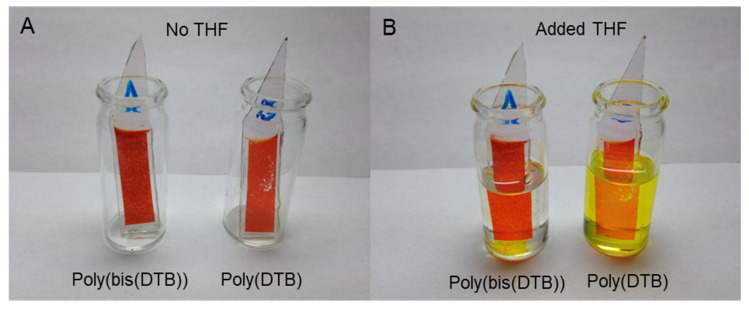
Solvent resistance of poly(DTB) and poly(bis(DTB)) to THF. (**A**) Before addition of THF; (**B**) after addition of THF.

Most polymer films obtained via electrochemical polymerization in LCs are insoluble in organic solvents both in their oxidized state and after reduction (dedoped). However, the monomer **DTB** displays a unique property where the corresponding polymer film has solubility in THF after reduction with hydrazine. This property enables a closer examination of the effect of crosslinking on the solvent resistance. As shown in Figure 8, the **poly(DTB)** film was partly dissolved in THF. **Poly(DTB)** in THF solution was yellow, while **poly(bis(DTB))** in THF solution was transparent, even though a small amount of yellow precipitate appeared. This contrast demonstrates that the crosslinking effect can improve the resistance to solvents in polymer films.

## 4. Conclusions

In this research, a fabrication method for forming conjugated polymer films was developed using electrochemical polymerization in LCs. By employing the monomers with crosslink properties and magnetic orientation, the conjugated polymer films were fabricated with both a crosslinked network structure and a uniaxial main chain orientation. Two types of monomers, uncrosslinked monomers and crosslinked monomers, were employed for the electrochemical polymerization in LCs, and a magnetic orientation was performed to obtain a uniaxially oriented LC electrolyte solution for the polymerization. The resulting polymer films showed a uniform optical axis. This result indicates that the conjugated polymer network possesses a uniaxially oriented conjugated main chain. An increase in the proportion of crosslinked monomers resulted in a decrease in the orientation degree for the main chains in the polymer. Moreover, changes in the alkyl side chains length of the monomers induced a significant change in the orientation direction of the main chains in the resultant polymers. The crosslinking is beneficial in improving the durability for conjugated polymers.

## 5. Instruments

^1^H NMR spectra were recorded on a JNM-ECS-400 NMR Spectrometer (JEOL, Tokyo, Japan). All signals were recorded in ppm with the internal TMS (tetramethylsilane) at 0.00 ppm. FT-IR absorption spectra were obtained with an FT/IR-4600 (JASCO, Tokyo, Japan) using the KBr method. All spectra were recorded between 400 and 4000 cm^−1^ with a resolution of 4 cm^−1^. POM observations were conducted with an Eclipse LV100 high-resolution polarizing microscope (Nikon, Tokyo, Japan). UV-vis absorption spectra were recorded on a V-630 UV-vis spectrophotometer (JASCO, Tokyo, Japan).

## Figures and Tables

**Table 1:** Concentration of each component of the electrolyte solution and the electrochemical polymerization conditions.

Component	Liquid Crystal	Supporting Salt	Reaction Units
Molar concentration	99%	0.50%	0.50%
Reaction time	15 min		
Voltage	4 V direct current		
Magnetic field intensity	12 T		
Temperature	rt.		
Electrode substrate	ITO glass		
